# Thymic Epithelial Cells Contribute to Thymopoiesis and T Cell Development

**DOI:** 10.3389/fimmu.2019.03099

**Published:** 2020-01-31

**Authors:** Hong-Xia Wang, Wenrong Pan, Lei Zheng, Xiao-Ping Zhong, Liang Tan, Zhanfeng Liang, Jing He, Pingfeng Feng, Yong Zhao, Yu-Rong Qiu

**Affiliations:** ^1^Laboratory Medicine Center, Nanfang Hospital, Southern Medical University, Guangzhou, China; ^2^State Key Laboratory of Membrane Biology, Institute of Zoology, Chinese Academy of Sciences, Beijing, China; ^3^Department of General Surgery, Taihe Branch of Nanfang Hospital, Southern Medical University, Guangzhou, China; ^4^Division of Allergy and Immunology, Department of Pediatrics, Duke University Medical Center, Durham, NC, United States; ^5^Department of Urological Organ Transplantation, Center of Organ Transplantation, The Second Xiangya Hospital of Central South University, Changsha, China

**Keywords:** thymic epithelial cells (TECs), medullary thymic epithelial cells (mTECs), thymopoiesis, tissue-restricted antigens (TRAs), tolerance

## Abstract

The thymus is the primary lymphoid organ responsible for the generation and maturation of T cells. Thymic epithelial cells (TECs) account for the majority of thymic stromal components. They are further divided into cortical and medullary TECs based on their localization within the thymus and are involved in positive and negative selection, respectively. Establishment of self-tolerance in the thymus depends on promiscuous gene expression (pGE) of tissue-restricted antigens (TRAs) by TECs. Such pGE is co-controlled by the autoimmune regulator (Aire) and forebrain embryonic zinc fingerlike protein 2 (Fezf2). Over the past two decades, research has found that TECs contribute greatly to thymopoiesis and T cell development. In turn, signals from T cells regulate the differentiation and maturation of TECs. Several signaling pathways essential for the development and maturation of TECs have been discovered. New technology and animal models have provided important observations on TEC differentiation, development, and thymopoiesis. In this review, we will discuss recent advances in classification, development, and maintenance of TECs and mechanisms that control TEC functions during thymic involution and central tolerance.

## Update on TEC Classification

**Figure 1:**
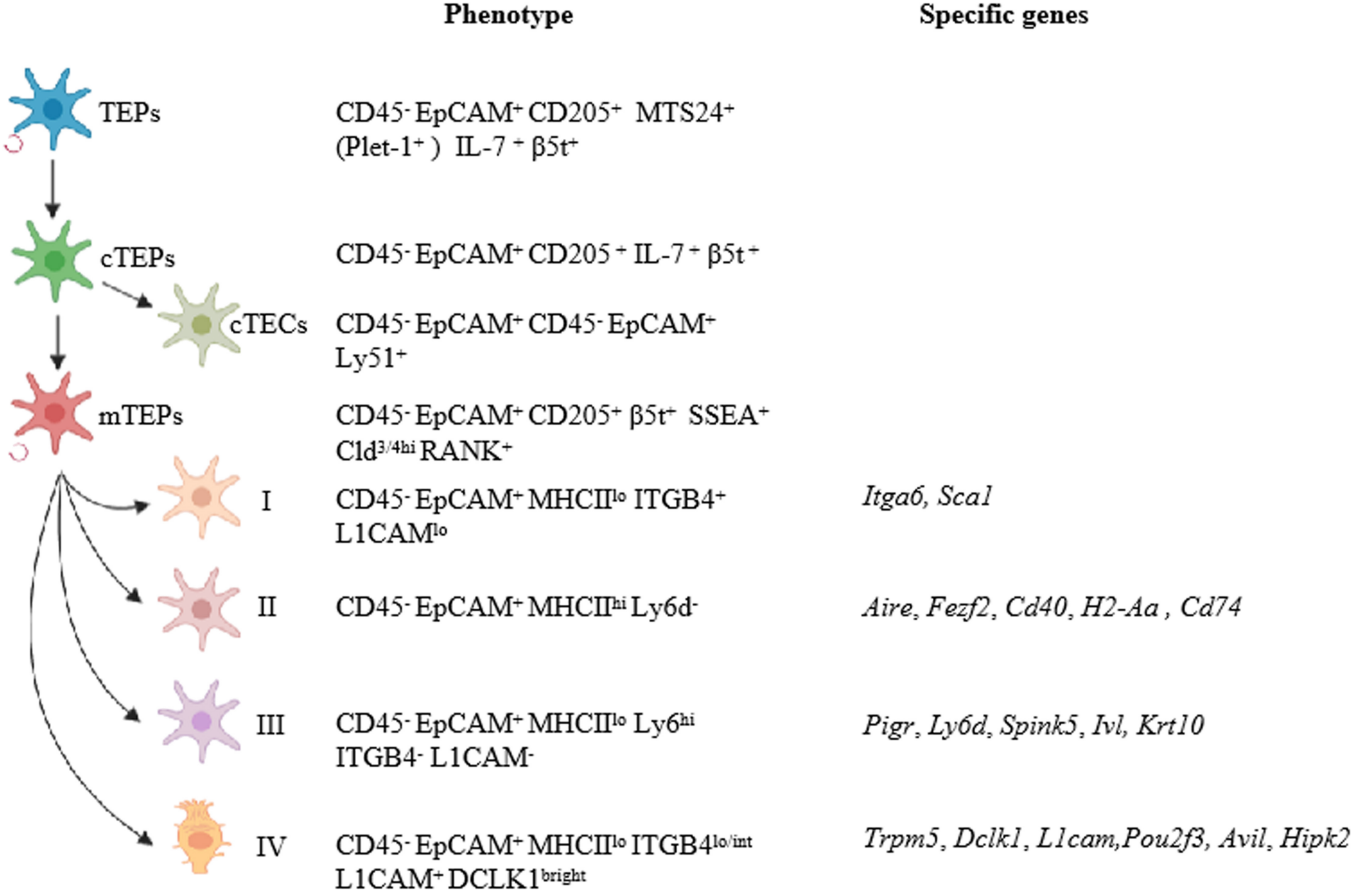
Updates on TEC classification. TECs can be separated into cortical and medullary TECs based on their localization in the thymus. Bipotent TEC progenitors with the ability to self-renew were identified as EpCAM^+^UEA1^−^Ly-51^+^MHCII^hi^Plet1^+^ in adult thymus. cTEPs are positive for EpCAM, CD205, IL-7, and β5t. cTECs that differentiated from cTEPs are defined as EpCAM^+^Ly-51^+^CD45^−^, whereas mTECs are characterized by EpCAM^+^UEA-1^+^CD45^−^. mTEC compartments were newly reclassified into four major subsets, termed mTEC I–IV. mTEC I is EpCAM^+^CD45^−^MHCII^lo^ITGB4^+^L1CAM^lo^ and usually expresses *Itga6* and *Sca1*. The mTEC II population is considered to be mature mTECs and is defined as EpCAM^+^CD45^−^MHCII^hi^Ly6d^−^. mTEC II cells express *Aire, Fezf2, CD40, H2-Aa*, or *CD74*. The mTEC III population is EpCAM^+^CD45^−^MHCII^lo^Ly6d^hi^ITGB4^−^L1CAM^−^ and expresses *Pigr, Ly6d, Spink5, Ivl*, and *KRT10*. Remarkably, the mTEC IV population expresses high levels of L1CAM and DCLK1 and a specific set of genes, such as *Trpm5, Dclk1*, and *L1cam*, but with low levels of MHCII.

The thymus is the primary lymphoid organ responsible for the generation and maturation of T cells. Thymic epithelial cells (TECs), the most abundant cell population within the thymic stroma, are further separated into cortical and medullary TECs (cTECs and mTECs) based on their localization within the thymic cortex or medulla, respectively. cTECs are essential for the positive selection of T cells, while mTECs play a major role in inducing negative selection of highly self-reactive T cells needed for establishing central self-tolerance (1). Generally, cTECs are defined as EpCAM^+^Ly-51^+^CD45^−^ by flow cytometry and keratin 8 (KRT8) expression, whereas mTECs are characterized by a reactivity with Ulex europaeus agglutinin I (UEA-1), defined as EpCAM^+^UEA-1^+^CD45^−^, and are positive with keratin 5 (KRT5). In an embryonic thymus, cTECs are the dominant population, while mTECs account for most of the TECs in the adult thymus. mTECs can be broadly subdivided into two distinguishable subsets in the adult murine thymus [immature major histocompatibility complex (MHCII)^low^CD80^low^ (mTEC^lo^) and mature MHCII^high^CD80^high^ (mTEC^hi^)], according to the expression levels of several maturation molecules, such as MHCII and CD80. mTEC^lo^ cells can serve as precursors for mature mTEC^hi^ cells. Moreover, the Aire has also been suggested as a marker of mature mTECs (2, 3). Mature mTECs are identified by surface markers CD205^−^RANK (receptor activator of nuclear factor-κB)^+^LTbR^+^MHCII^hi^Aire^+^ in the embryonic thymus and are defined as Ly51^−^UEA-1^+^RANK^hi^MHCII^hi^Aire^+^ in the adult. Using unsupervised single-cell RNA-sequencing and fate-mapping analyses, the mTEC compartment was recently reclassified into four major subsets, termed mTEC I–IV (4). These subsets have distinct transcriptional and molecular characteristics. Specifically, mTEC I is EpCAM^+^CD45^−^MHCII^lo^ITGB4^+^L1CAM^lo^ and usually expresses Itga6 and Sca1. The mTEC II population is considered to be mature mTECs and is defined as EpCAM^+^CD45^−^MHCII^hi^Ly6d^−^. mTEC II cells express Aire, Fezf2, CD40, H2-Aa, or CD74. The mTEC III population is EpCAM^+^CD45^−^MHCII^lo^Ly6d^hi^ITGB4^−^L1CAM^−^ and expresses Pigr, Ly6d, Spink5, Ivl, and KRT10. Notably, the mTEC IV population, referred to as EpCAM^+^CD45^−^MHCII^lo^ITGB4^lo/int^L1CAM^+^, is also detected as DCLK1^bright^ and expresses a specific set of genes, such as Trpm5, Dclk1, and L1cam, but does not express any classical mTEC or cTEC markers (Figure 1). At the age of embryonic day 18.5 (E18.5), mTEC I and II subpopulations become detectable and can be the most proliferative cells in the TECs. The mTEC III can be found in the 4-week-old thymus. On neonatal day 6, mTEC IV cells are present and represent a putative tuft cell type by comparing their transcriptional profile to intestinal tuft cells and to the different TEC populations. These tuft cells account for 5–10% of total mTECs in the thymus and share a large number of regulatory factors with tuft cell–specific genes, including Avil, Il25, Hipk2, and Pou2f3 (4, 5). Moreover, mice with either a Pou2f3 deficiency (Pou2f3^−/−^) or a TEC-specific deletion of Hipk2 have blocked differentiation of these tuft cells. Thymic tuft cells are characterized by high and exclusive expression of IL-25 and are critical for the development and intrathymic function of type 2 invariant natural killer T (NKT2) cells, which are the dominant source of interleukin-4 (IL-4) in the medulla (4, 5). A new analysis of thousands of single mTECs revealed 15 subpopulations. Consistent with the data from Bornstein et al., clusters 1 and 2 relate to the immature mTEC I subpopulation; clusters 3 and 6, to mature mTEC II; clusters 7 and 8, to mTEC III; and cluster 10, to the tuft-like mTEC IV (6). Further studies should focus on the functions of these newly defined mTEC and cTEC subsets in thymopoiesis, thymic involution, and T cell development.

## Characterization of TEC Progenitors

TECs have dual germ layer origins, with cTECs and mTECs arising from ectoderm and endoderm, respectively (7). There are also studies showing that cTECs and mTECs share a common endodermal origin (8). The fetal bipotent TEC progenitors (TEPs), defined as Plet-1^+^ (using the monoclonal antibody MTS24), CD205^+^, IL-7^+^, and β5t^+^, have been shown to generate both cTECs and mTECs (9–13). It was reported that MHCII^hi^Plet-1^+^Ly-51^+^ TECs include bipotent progenitors that can generate both mTECs and cTECs, while MHCII^lo^Plet-1^+^Ly-51^+^ TECs may contribute to only cTECs. TEPs have been shown to have self-renewal capacity (14). It remains unclear whether the bipotent TEPs or unipotent precursors of TECs drive the differentiation of cTECs. However, the development and maturation of mTECs are better understood. As reported by Hamazaki et al. in the early embryonic thymus, the tight junction components Claudin3^+^ and Claudin4^+^ (Cld3/4^+^) mTEPs represent the initial progenitors that exclusively generate mTECs but not cTECs (15). A minor SSEA-1^+^ fraction within the embryonic as well as adult Cld3,4^hi^ TECs was shown to generate mature mTECs. However, SSEA-1^+^Cld3,4^hi^ mTECs in adults show markedly reduced potential of generating mature mTEC progenies because of concomitant active thymopoiesis (16). Comparable with keratinocytes, mTECs have high turnover rates in a matter of weeks (2). It has been reported that the entire TEC population has a close to 10% turnover rate per day, which occurs predominantly within the Aire^−^mTEC^hi^ subset (17). TECs are largely EpCAM^+^CD45^−^${\text{Sca-}1}^{-}\alpha_{6}$integrin^lo^ at E15; however, the EpCAM^+^CD45^−^${\text{Sca-}1}^{\text{hi}}\alpha_{6}$integrin^hi^ TEC subset has been shown to persist into adulthood. These cells display stem/progenitor potential and give rise to downstream mTEC^lo^ and mTEC^hi^ subsets (18). Ulyanchenko et al. identified EpCAM^+^UEA1^−^Ly-51^+^MHCII^hi^Plet1^+^ cells as bipotent TEC progenitors in adult thymus (14). Therefore, TEPs appear in embryonic, postnatal, and even adult thymus. Further resolution of the postnatal bipotent epithelial progenitor phenotype and understanding of its role in postnatal TEC maintenance is critical.

## TEC and Thymic Involution: Transcription Factors and Signaling

The Forkhead box protein N1(Foxn1) is the most important transcription factor expressed in both cTECs and mTECs. It regulates the development, differentiation, and function of TECs both in the fetal and adult thymus (19–21). In the absence of Foxn1, expression of the genes encoding the chemokines Ccl25 and Cxcl12 as well as the cytokine Scf (also known as Kit ligand) cannot be detected in the Foxn1-deficient thymic epithelium (22). Moreover, functional loss of Foxn1 causes athymia, whereby TECs cannot progress beyond an immature phenotype or attract hematopoietic precursor cells. In the nude thymus anlage, TECs may fail to express cell-surface molecules involved in the adhesion of hematopoietic progenitor cells to epithelial cells (23). It was reported that Foxn1 transgenic mice can rescue the nude thymus phenotype (24). Foxn1Cre mice containing an IRES-Cre cassette inserted into the 3′ untranslated region in the Foxn1 locus to direct Cre expression starting on embryonic day 11.5 in TECs (25) are widely used to explore the function of genes after specific deletion in thymic epithelial cells. Additionally, a BAC transgenic model is also a useful tool to express genes of interest in TECs (26, 27).

It is important to note that Foxn1 is also expressed in the hair follicle and is crucial for hair follicle development. Disruption of Foxn1 in mice results in not only severe thymic atrophy but also the hairless (nude) phenotype (24). Most recently, Larsen et al. reported that a knockout of regions of the first intron of Foxn1 severely disrupts thymus development but causes no defects in the skin, suggesting that distinct cis-regulatory elements control Foxn1 expression in TECs and hair follicles (28). In addition, a new mutant of Foxn1 (Foxn1^Δ/Δ^) in mice, with most of its N-terminal domain truncated, caused abnormal thymic architecture, including absence of the cortical and medullary regions, without apparent defects in the skin and hair (29). Thus, despite its crucial role in both TECs and hair follicles, distinct mechanisms may regulate its expression and function in these cells.

Thymocyte-derived signals are required to sustain TEC maturation, and the molecular nature of the bidirectional links between thymocytes and TECs have also been identified. TEC organization and maturation is dependent on or regulated by thymocyte-derived signals. Rag2^−/−^ mice, which lack mature thymocytes, are defective in thymopoiesis and have poor development of the medulla and cortex (30). cTEC maturation relies on signals provided by double negative (DN) and double positive (DP) immature thymocytes, whereas mTEC maturation depends on signals provided by single positive (SP) mature thymocytes (31). Such lymphostromal interaction, which is called “thymus crosstalk,” between developing lymphocytes and epithelial cells contributes to thymus development (32). In addition, as ligand-receptor signals, TECs can efficiently acquire not only cell surface but also intracellular proteins from thymocytes (33). Whether such acquisition of protein components from thymocytes by TECs impact their development and/or function remains to be defined.

## NF-κB Signaling Pathways

Multiple studies have revealed that both the canonical and non-canonical NF-κB signaling pathways are indispensable for the development of mTECs and their progenitors (34–36). The canonical NF-κB pathway can be activated by TNF receptor associated factor 6 (TRAF6), which is downstream of TLRs (37). Other TNF receptor superfamily (TNFRSF) members, including CD40, lymphotoxin β receptor (LTβR), and receptor activator of nuclear factor-κB (RANK, also known as Tnfrsf11a), are expressed in mTECs and also involved in NF-κB activation (36, 38, 39). Mature SP thymocytes are the main source of LTβR ligands. Absence of LTα1β2, an LTβR ligand, causes abnormal differentiation of mTECs in mice (40). Mice with RANK ligand (RANKL) or CD40 deficiency (Tnfsf11^−/−^ or CD40^−/−^) show normal thymic architecture and a slightly decreased mature mTEC number, while double deficiency (Tnfsf11^−/−^CD40^−**/**−^) impairs the postnatal development of mTECs (36). Consistently, data from CD40 ligand-deficient (CD40L, a TNF superfamily cytokine produced by positively selected thymocytes) mice (CD40lg^−/−^) revealed normal medullar organization but decreased mTEC^hi^ cells in the thymus (2, 36). Moreover, Tnfrsf11a^−/−^ mice exhibit a severe reduction in mature mTECs in the adult; however, no defect in medullary organization or development of KRT5^+^ mTECs was observed in Tnfrsf11a^−/−^ mice (41). RANKL produced by positively selected thymocytes medicates thymic crosstalk. RANKL and its decoy receptor, osteoprotegerin (OPG), can regulate the mTEC cellularity and formation of thymic medulla that contains Aire-expressing mTECs (42).

Data from a fetal thymus organ culture (FTOC) suggested that RANKL and CD40L signal through their receptors to activate TRAF6 and NF-κB-inducing kinase (NIK) to induce the expression and activation of RelB (36). NIK, IκB kinase (IKK) α, and RelB are also essential for mTEC development (30, 34, 43, 44). In addition, Shen et al. reported that specific deletion of NIK and IKKα can also lead to severe autoimmune hepatitis, liver injury and fibrosis, and lung autoimmune disease (45). In conclusion, both the canonical and non-canonical NF-κB pathways that integrate signals from multiple TNF superfamily receptors contribute to not only TEC maturation but also thymus development.

## Wnt Signaling

Wnt proteins, constituting a large group of secreted glycoproteins, are implicated in a wide variety of biological processes, including cell survival, proliferation, migration, and differentiation (46). Balciunaite et al. have found that Wnt glycoproteins are expressed by TECs and thymocytes and only certain Wnt proteins control Foxn1 transcription in TECs via Akt phosphorylation (47). Both Wnt1 and Wnt4 were able to signal to TECs (47, 48). The number of thymocytes was reduced by 20–30% in Wnt-1^−/−^ or Wnt-4^−/−^ mice and 50–70% in Wnt-1 and Wnt-4 double-deficient (Wnt-1^−/−^Wnt-4^−/−^) fetal thymus, suggesting that these two molecules perform a redundant, or synergistical, role in TECs. Moreover, mice lacking Wnt-1 and/or Wnt-4 die at birth, limiting the analysis of adult thymocyte maturation in these mice (49). In general, Wnt signals function through β-catenin-dependent and β-catenin-independent pathways (47). β-catenin, encoded by the Ctnnb1 gene, is a dominant factor of classical Wnt signaling, while NLK is an effective regulator of the β-catenin-independent pathway (50–52). Although, the total number of thymus was reduced by nearly 50% in newborn Ctnnb- and NLK-deficient mice, the normal histological phenotypes in these mice on E15.5 suggest that the canonical Wnt signaling in TECs is not crucial, at least in the early stage of thymopoiesis (53). However, TEC-specific β-catenin-deficient mice die within a few days of birth due to defects in skin development, which limits examination of this pathway in the adult thymus (53). Interestingly, TEC-intrinsic overexpression of β-catenin causes defective thymic total cell number as well as TEC cellularity, indicating that proper Wnt signaling is important for normal thymic development (53).

In addition, Gpr177 is a Wnt-specific cargo receptor that is essential for Wnt-ligand transportation and Wnt secretion (54, 55). Data from Gpr177^f/f^Foxn1Cre mice show that TECs are unable to secrete Wnt ligands, which leads to a reduction in the thymus size and total number of thymocytes as well as a peripheral T cell pool (56). Several recent papers have described that the bone morphogenetic protein (BMP) family, in particular BMP4, regulates Foxn1 expression and early TEC differentiation during aging (57–59). Rossi et al. reported that the keratinocyte growth factor (KGF) signaling pathway in TECs activates p53 and NF-κB pathways and leads to the transcription of several target genes required for TEC function and T cell development, including BMP2, BMP4, Wnt5b, and Wnt10b (60). Inhibition of the BMP signals through BMP antagonist Noggin results in the formation of dysplastic thymic lobes of drastically reduced size (57), a phenotype similar to that seen in mice with activated Wnt signaling. Overall, these data demonstrate that specific Wnt signaling is essential for TEC development and thymopoiesis.

## mTOR Signaling

The serine/threonine kinase mammalian/mechanistic target of rapamycin (mTOR), which signals through two complexes, mTORC1 and mTORC2, controls cell growth, proliferation, autophagy, and metabolism (61). Recently, mTOR knockout mice (mTOR^f/f^Foxn1Cre), which affect both mTORC1 and mTORC2, were also found to affect thymopoiesis as well as the development and maintenance of TECs, in particular mTECs. Additionally, mTOR regulates Wnt-signaling activity through autophagy, which is crucial for TEC development and maturation (62). A study stated that phospho-P70s6k is increased in all thymomas, but not in normal thymus, and the activation of the mTOR/Akt pathway is involved in the pathogenesis of thymic epithelial tumors (63).

We have revealed that mTORC1/Raptor signaling in TECs is crucial for thymopoiesis. Proper generation of multiple T cell lineages, including Tregs, iNKT cells, γδT17 differentiation, as well as TCRVγ5/6Vδ1 recombination, was affected in TEC-specific, Raptor-deficient mice. These mice display severe thymic atrophy, anomalous thymic architecture, and decreased mTEC/cTEC ratios (64). Furthermore, we also reported that TEC-specific deletion of mTORC2/Rictor causes moderate thymic atrophy, with a 44.6–33.9% decreased total thymic cellularity from neonate to 3 months old. Moderately reduced mTEC numbers and production of virtually all T cell lineages in the thymus compare with mTORC1/Raptor-deficient mice. However, iNKT cells appear to be more severely reduced in Rictor/mTORC2-deficient mice (65). Nevertheless, future studies should focus on the downstream mechanisms of mTOR or mTORC1/2 in TECs and their role in thymopoiesis.

## Other Signaling Involved in TEC Development

In addition to the aforementioned signal pathways and transcription factors, other signaling pathways contribute to TEC development and function. Signal transducer and activator of transcription 3 (Stat3) is a crucial regulator of several cellular processes, including cell growth, survival, and differentiation (66). Using Stat3^f/f^-KRT5Cre mice with Stat3 deleted in the epithelia of the skin, also known as the epidermis, showed thymic hypoplasia and accelerated thymic aging, indicating that Stat3 is critical for maintaining thymic architecture and for thymocyte survival (67). Contrary to this finding, a study from Satoh et al. showed that a Stat3-meditated signal via EGF-R is required for the postnatal development of thymic medullary regions (68). Meanwhile, similar results were found, which showed using gain- and loss-of-function genetic approaches to prove that Stat3 signaling plays a vital role in mTEC development and maintenance, rather than its proliferation (69). Collectively, these results showed that Stat3 signaling plays important roles in mTECs but not in cTECs. Interestingly, mTORC1 can directly phosphorylate Stat3 at Ser727 to promote transcriptional activity (70). Whether Stat3 phosphorylation and function are affected in mTORC1-deficient TECs to contribute to the abnormal phenotypes in these mice remains to be determined.

Mesenchymal epithelial transition factor (c-Met) is a multifunctional transmembrane tyrosine kinase and acts as a receptor for hepatocyte growth factor (HGF) (71). c-Met is expressed by thymocytes, TECs, and early T progenitors, each of which is capable of regulating thymopoiesis (72). A c-Met-specific deficiency in TECs results in age-progressive reduction in TEC number and a reduced number of regulatory T cells. Consequently, c-Met TEC-conditional knockout mice displayed an autoimmune phenotype due to the severe infiltration of lymphocytes into multiple organs and increased activity of autoantibodies to peripheral tissue antigens (73). Thus, c-Met signaling in TECs is important for the maintenance of TECs and immune self-tolerance.

Myc proteins are another transcription factor found to be important in TECs. Myc levels are closely related to cell proliferation and affect cell-cycle progression (74). Myc^f/f^Foxn1Cre mice show 50–65% decreases of absolute number of both cTECs and mTECs. The reduced TEC numbers cause a smaller yet normally structured thymus that supports normal thymocyte differentiation, suggesting that c-Myc expression in TECs is essential for the proliferation of both mTECs and cTECs but is not required for the maturational progression of mTECs (75). Interestingly, Myc activity declines in TEC during development and TEC-specific transgenic expression of Myc increased the adult thymic size, numbers of active ribosomes, and the frequency of proliferating TEC (76).

TGF-β also participates in thymopoiesis. It is a potent regulator of cell-cycle progression, development, and differentiation. The TGF-β signaling pathway has recently been found to be upregulated in the fetal thymi (77). TGF-β may limit the size of the mTEC compartment, particularly affecting the MHCII^hi^ population. A specific lack of TGF-β signaling in TECs leads to accelerated thymopoiesis after irradiation (78). TGF-β signaling may negatively modulate signals required for mTEC differentiation and maturation and inhibit anti-CD40–induced non-canonical NF-κB signaling (75).

Histone deacetylases (HDACs) play a crucial role in controlling cell-cycle regulation and differentiation and tissue development (79). Conditional deletion of either HDAC1 or HDAC2 only slightly affects thymic size and TEC cellularity. However, inactivation of HDAC3 causes obvious thymic atrophy, with impaired development and maturation of mTECs but without affecting the cTECs. Moreover, HDAC3-mediated repression of Notch in TECs is required for efficient TEC/mTEC development (80). It remains to be determined whether downregulated Notch activity is responsible for the abnormal mTEC phenotypes in HDAC3-deficient mice.

Sonic Hedgehog (Shh), one of three mammalian Hedgehog proteins expressed in the thymus, can regulate T cell development (81, 82). In the mice with a TEC-specific loss of Shh expression, fetal thymus atrophy and numbers of both cTEC and mTEC lineage cells were significantly reduced, whereas the maturation molecular MHCII was increased on both mTEC and cTEC subsets (83). Gli3 is a transcription suppressor that inhibits Shh transcription (84). Gli3-deficient mice have increased TECs, but these TECs express decreased MHCII molecules (83). TEC-specific Gli3-deficient mice show a significant reduction of CD4 single positive cells and double positive thymocytes and a concomitant increase of the DN population in the fetal thymus. These results suggest that Gli3 expressed in TECs promotes positive selection and T cell maturation by suppression of Shh expression in TECs (85).

Chromobox homolog 4 (Cbx4) is not only a member of the Polycomb group (PcG) family, which is highly expressed in the TECs, but is also a downstream factor of p63 (as a marker for putative epithelial progenitors), which regulates TEC development (86, 87). TECs specifically deficient of Cbx4 exhibited impaired proliferation and maturation. Cbx4 modulates T lymphopoiesis by regulating the proliferation of TECs and the maintenance of the thymic epithelium (87). Furthermore, Foxa1 and Foxa2 are widely co-expressed during murine embryogenesis and in adult epithelium tissues and are required for the establishment of competence within the foregut endoderm and at the onset of hepatogenesis (88, 89). Conditional deletion of Foxa1 and Foxa2 from TECs causes a smaller thymus and an increase in the mTEC percentage and Aire expression but lower MHCII expression on Aire^+^ mTEC subsets. Both Foxa1 and Foxa2 are necessary for normal TEC differentiation and function and have important effects on T cell development and regulation of T cell selection (90). Currently, many signaling pathways and transcription factors involved in the development and maturation of mTECs have been identified. Future studies may explore cross-regulation of these signal pathways and transcription factors and identify signaling and transcriptional programs that govern cTEC differentiation and maturation.

## Thymic Involution

Infection, aging, pregnancy, stress, and other processes can all cause thymic atrophy, or involution, resulting in decreased thymus production and outward migration of naïve T cells and increasing susceptibility to infections (48, 91–93). In addition, some agents, including cyclosporine, cyclophosphamide, and dexamethasone treatments, can also affect the growth and development of TECs, leading to thymus atrophy (94). Analysis of RNA-seq of the transcriptional profiles of mTEC from 2-, 6-, and 10-week-old mice indicated diminished expression of cell cycle-related genes and E2F3 target genes but increased expression of inflammatory chemokines and cytokines along with upregulated TNFα signaling, CD40 signaling, and RANK signaling during the early phase of thymic involution (95). MicroRNAs, such as miR-183-5p, miR-199b-5p, miR-205-5p, and miR-200b-3p, and their target genes may serve as biomarkers for mouse thymus development and involution (96). Recently, TEC exosomes and MiR27b have been linked with thymus tissue regeneration and thymic adipose involution (97). Molecular mechanisms involved in thymopoiesis are most focused on the transcription level. However, protein-level mechanisms controlling thymic involution and TEC homeostasis are rarely reported. A study by St-Pierre et al. presented that the rate of protein synthesis of mTEC^hi^ subsets was nearly twice that of thymocytes. They also reported that most pGE occurs in the more differentiated mTEC^hi^ subset and that immunoproteasome (IP, which can regulate cell-cycle progression, differentiation, development, and morphogenesis) deficiency resulted in lower TEC cellularity, particularly in mTECs but not in cTECs. Lack of IPs in mTECs resulted in autoimmune manifestations, defective regeneration capacity in adult mTECs, and severe proteotoxic stress in the cells (98).

Psmb11, a type of thymoproteasome, is a third catalytic chain unique to cTECs. Most recently, a study from Apavaloaei et al. presented that Psmb11 can regulate the differentiation of cTECs mainly by repressing Wnt signaling. Interestingly, the expression profile of chemokine and claudin genes in Psmb11^−/−^ cTECs offered noticeable likenesses to those of wild-type mTECs, suggesting that, in the absence of Psmb11, cTECs acquire features of mTECs (99). Most recently, in combination with transcriptomic analysis, transomics analysis revealed a highly specific impact of β5t on proteasomal subunit composition in cTECs, indicating that a β5t-containing thymoproteasome governs CD8^+^ T cell development (100). Cellular architectural proteins often contribute to organ growth and maintenance. Lamin-B1 is a structural protein of the nuclear lamina that forms a filamentous meshwork in the nucleus. This protein was reported to be reduced in the aging thymus. TEC senescence and TEC lamin-B1 reduction can be triggered by several proinflammatory cytokines. Moreover, ablation of Lmnb1 (lamin-B1 gene) in TECs caused a significant decrease in the size and total cell number in the 2-month-old mouse thymuses, indicating that Lamin-B1 is critical for TEC development and maintenance and proper thymic involution (101). Recently, a study by Venables et al. reported that during the process of thymic atrophy and regeneration, the morphology of cortical epithelium is controlled by signals from the medullary stroma, and the thymus size is mainly regulated by the rate-limiting morphological changes in the cortical stroma, rather than by their cell death or proliferation (102). Because thymic degeneration is a dynamic process, it is challenging to explore the mechanism of thymic involution.

## Promiscuous Gene Expression and Immune Tolerance

Accumulating evidence suggests that pGE of TRAs in mTECs is essential to induce tolerance (103, 104). The transcriptional regulator Aire is selectively expressed in mTECs where it plays a key role in promoting pGE of a wide array of TRAs (105–107). Consistent with previous reports, live-cell imaging has revealed that distinct dendritic cell (DC) subsets in the thymus and donation of diverse self-antigens from Aire^+^ mTECs to these DCs for antigen presentation are essential for establishing central tolerance (108). Although most TRAs are induced by Aire, some TRAs are also upregulated in mature mTECs in the absence of Aire (103). It has been suggested that the Fezf2 is responsible for Aire-independent TRA expression in mTECs (109). Using a high-throughput, single-cell, RNA-seq approach, Kernfeld et al. indicated that expression of autoimmune-implicated genes may begin during embryogenesis (110). Aire expression was regulated by members of the TNF-receptor superfamily, such as Tnfrsf11a and CD40 (104). Aire-deficient (Aire^−/−^) mice have altered thymic morphological and distribution of mTECs together with a manifestation of autoimmune disease but without autoantibody production (111). Autoimmune Polyglandular Syndrome Type-I (APS-1/APECED) were also triggered by mutations in the Aire gene (112, 113). Aire also promotes the immune checkpoint HLA-G gene and increases distribution of intracellular HLA-G protein expression in TECs (114). Furthermore, a previous study showed that CD4^+^CD3^−^ lymphoid tissue inducer (LTi) cells were involved in inducing Aire, CD80, and Aire-dependent TRA expression in mTEC development by producing the cytokine RANKL (41). Liang et al. described that TEC-specific deletion of mTOR can also lead to the development of severe autoimmune diseases by regulating Aire-dependent TRA gene expression (62). The work by Takaba et al. further indicated that mice lacking Fezf2 in mTECs exhibited severe autoimmune symptoms, including the production of autoantibodies and inflammatory cell infiltration targeted to peripheral organs (109). Fezf2 function is mostly dependent on the lymphotoxin (LT) β-signaling axis but not the RANK/CD40-Aire axis (115). Both Aire-dependent expression and Fezf2-dependent TRA expression in mTECs are important for central tolerance. Impairment of either of them can lead to loss of self-tolerance and autoimmunity.

## Conclusion

Recent studies have improved the understanding of central roles for TECs in thymocyte development and established that crosstalk between developing thymocytes and TECs ensures proper TEC differentiation and development, which impacts thymopoiesis. Despite recent advances, several features of TEC development and physiology remain uncharacterized. They include the developmental mechanisms that drive differentiation of immature progenitors into mature cTECs, DC, and mTEC regulation role for both negative selection and Treg development, the exact mechanisms and crucial pathways that control age-related thymic involution, as well as stress-related thymic involution. An effective TEC isolation and 3D culture strategy needs to be developed. There is also an urgent need to develop safe therapies against immunosenescence to prevent thymic involution and a non-invasive strategy to restore thymus function in human patients with TEC dysfunction.

## Author Contributions

H-XW, WP, and LZ wrote the manuscript. X-PZ reviewed and edited the manuscript. LT, ZL, JH, and PF edited the manuscript. YZ and Y-RQ provided direction and edited the manuscript.

### Conflict of Interest

The authors declare that the research was conducted in the absence of any commercial or financial relationships that could be construed as a potential conflict of interest.
