# Corrigendum: Thymic Epithelial Cells Contribute to Thymopoiesis and T Cell Development

**DOI:** 10.3389/fimmu.2020.628464

**Published:** 2020-11-30

**Authors:** Hong-Xia Wang, Wenrong Pan, Lei Zheng, Xiao-Ping Zhong, Liang Tan, Zhanfeng Liang, Jing He, Pingfeng Feng, Yong Zhao, Yu-Rong Qiu

**Affiliations:** ^1^ Laboratory Medicine Center, Nanfang Hospital, Southern Medical University, Guangzhou, China; ^2^ State Key Laboratory of Membrane Biology, Institute of Zoology, Chinese Academy of Sciences, Beijing, China; ^3^ Department of General Surgery, Taihe Branch of Nanfang Hospital, Southern Medical University, Guangzhou, China; ^4^ Division of Allergy and Immunology, Department of Pediatrics, Duke University Medical Center, Durham, NC, United States; ^5^ Department of Urological Organ Transplantation, Center of Organ Transplantation, The Second Xiangya Hospital of Central South University, Changsha, China

**Keywords:** thymic epithelial cells (TECs), medullary thymic epithelial cells (mTECs), thymopoiesis, tissue-restricted antigens (TRAs), tolerance

In the original article, there was an error in the Funding statement. The correct numbers for National Nature Science Foundation of China are 81801390 to H-XW and 31800754 to ZL.

The authors apologize for this error and state that this does not change the scientific conclusions of the article in any way. The original article has been updated.

